# Reply on “Hip fracture and type 2 diabetes mellitus”: Bi-directional relationship between osteoporosis and diabetes

**DOI:** 10.1016/j.afos.2024.11.004

**Published:** 2024-12-04

**Authors:** Suhas Krishnamoorthy, Ching-Lung Cheung

**Affiliations:** Department of Pharmacology and Pharmacy, Li Ka Shing Faculty of Medicine, The University of Hong Kong, Hong Kong, China

We appreciate Doctor Seo's feedback on our publication [1]. Our study demonstrated a reduced diabetes risk in hospitalized fall patients with hip fractures compared to those without hip fractures [2]. Doctor Seo raised two main points: the omission of key factors like family history of diabetes, obesity, and body mass index (BMI), and the potential contradiction in showing a reduced diabetes risk in hip fracture patients given the known link between type 2 diabetes mellitus (T2DM) and higher hip fracture risk.

Regarding the adjustment of important factors, specifically family history of diabetes and BMI, we could not do so due to data limitations in the Hong Kong Clinical Data Analysis and Reporting System. Family history is unavailable, so the family history of diabetes could not be accounted for. However, to include a measure of adiposity in our study, obesity was included in our propensity score model, “Baseline characteristics of the real-world hip fracture cohort after matching”, that obesity was well matched between the exposed and unexposed groups with a standardized mean difference < 0.001.

Regarding the concern about the study's suggestion that hip fractures might reduce the risk of T2DM due to the known association between T2DM and increased hip fracture risk, this is an interesting argument. However, it's crucial to note the observational nature of our study, which limits the establishment of a causal relationship on this matter. Furthermore, as the relationship between bone and glucose metabolism is complex, the direction of association could be opposite in the bi-directional relationship between hip fracture and T2DM.

Our study acknowledges that T2DM is recognized in the literature as a well-documented risk factor for fractures. However, it is noteworthy to consider the paradoxical observation that individuals with T2DM often exhibit both higher bone mineral density (BMD) and an elevated fracture risk simultaneously. This phenomenon may be partially attributed to compromised bone microarchitecture in T2DM patients, which amplifies the risk of fractures despite their higher BMD levels [3]. To further investigate the relationship between bone and glucose metabolism, our previous study [4] using both cohort study and Mendelian randomization identified several key relationships: glycaemic traits displayed an inverse correlation with bone turnover and a positive association with BMD. Moreover, a higher BMD was linked to an increased risk of T2DM. Furthermore, through Mendelian randomization analysis, we demonstrated a genetic causal relationship where heel-estimated BMD was associated with higher 2-h glucose levels following an oral glucose challenge test and an elevated risk of T2DM. Building on these insights, we conducted a further study to evaluate the association of hip fracture with the risk of diabetes. This study indeed provided evidence to further dissect the relationship between bone metabolism and the risk of T2DM. Despite the increased risk of hip fractures in T2DM patients, these two studies showcased that lower BMD and hip fractures were associated with a reduced likelihood of developing T2DM. While we could not include important parameters like BMI and family history of diabetes in the hip fracture study, several important BMI- and metabolic-related variables were included in our previous, related study [4].

These studies overall provide new clinical insight into the role of bone metabolism on glycaemic control, aligning with an earlier animal study showing that osteoblasts are involved in regulating whole-body energy metabolism [5]. If our findings are further validated in other studies, it would be important to investigate how bone metabolism can regulate glucose metabolism in humans, which could provide novel insight into the management of bone fragility in diabetes.

## Conflicts of interest

The authors declare no competing interests.
